# Anti-NMDAR encephalitis with bilateral basal ganglia MRI lesions at a distance of time: a case report

**DOI:** 10.1186/s12883-022-02652-y

**Published:** 2022-03-28

**Authors:** Dong Keun Son, Seong Min Cho, Han Uk Ryu, Byoung-Soo Shin, Hyun Goo Kang

**Affiliations:** 1 Jeonbuk National University Medical School, Jeonju, South Korea; 2 Department of Neurology & Research Institute of Clinical Medicine of Jeonbuk National University - Biomedical Research Institute of Jeonbuk National University Hospital, 20 Geonji-ro, Deokjin-gu, Jeonju-si, Jeonbuk-do 54907 South Korea

**Keywords:** Anti-N-methyl-D-aspartate receptor encephalitis, Magnetic resonance imaging, Basal ganglia, Seizure

## Abstract

**Background:**

Approximately half (55%) of anti-N-methyl-D-aspartate receptor (NMDAR) encephalitis is known to show abnormal brain images, including high signal intensity in T2 or fluid attenuated inversion recovery (FLAIR) images. In a minority of anti-NMDAR encephalitis cases, high signal intensity on diffusion-weighted imaging (DWI) has been reported, a finding that is highly suggestive of a stroke.

**Case presentation:**

We present the case of a 66-year-old man who experienced two separate focal seizure events, which involved first the right and then the left upper extremity in a short period of time. The patient showed focal clonic seizures involving right arm and hand, which sometimes evolved to bilateral tonic-clonic seizures on his first admission. Brain magnetic resonance imaging (MRI) showed high signal intensity on DWI and low signal intensity on the apparent diffusion coefficient (ADC) map of the left caudate nucleus and putamen. The patient was discharged symptom-free with anti-epileptic drugs for 2 weeks. The second admission occurred 4 days after the discharge. He exhibited a new symptom of focal clonic seizures involving left arm and hand while showing a brain lesion on the opposite side which is hyperintense on DWI image and hypointense on ADC map. The patient was eventually diagnosed with anti-NMDAR encephalitis according to the cerebrospinal fluid (CSF) antibody test.

**Conclusions:**

This is the case of anti-NMDAR encephalitis patient whose DWI/ADC images revealed sequential involvement on the left and right basal ganglia with a short time interval. When stroke-like brain lesions on DWI are found in a patient with a focal seizure, a CSF study could help rule out autoimmune encephalitis. We also suggest that DWI/ADC map images may be useful for the early detection of anti-NMDAR encephalitis.

## Background

Anti-N-methyl-D-aspartate receptor (NMDAR) encephalitis is the most common type of autoimmune encephalitis. The major clinical features of the disease include psychosis, speech disorder, seizure, dyskinesia, decreased level of consciousness, and autonomic dysfunction. Patients can be diagnosed with anti-NMDAR encephalitis when meeting the following three criteria: 1) The patient presents with at least one of these symptoms. 2) Alternative causes are excluded. 3) Anti-NMDAR antibodies are detected in the cerebrospinal fluid (CSF) studies.

Approximately half (55%) of patients with anti-NMDAR encephalitis show abnormal brain images [1]. In most cases, increased signal intensity of the medial temporal lobe or cerebral cortex is reported in T2 images. Some case reports have shown hypermetabolism in ^18^F-fluorodeoxyglucose-positron emission tomography (^18^F-FDG-PET) images involving the insular cortex, hippocampus, basal ganglia, cerebellum, and brainstem, suggesting acute inflammation when correlative to MRI [2]. However, brain abnormalities are rarely found on diffusion-weighted imaging (DWI).

Here, we report a case of anti-NMDAR encephalitis with rare MRI findings. In this case, the patient was admitted twice for focal seizure events, first on the right side and second on the left. At each time of the seizure event, the contralateral basal ganglia showed high signal intensity on DWI and low signal intensity on apparent diffusion coefficient (ADC), which was indicative of acute ischemic stroke. However, the CSF antibody test revealed anti-NMDA receptor antibody and finally confirmed anti-NMDAR encephalitis.

## Case presentation

A 66-year-old man presented to our emergency department with a clonic seizure on his right arm and hand. The seizure lasted for 15 s at 30 s intervals, which then progressed to bilateral tonic-clonic seizures. He was diagnosed with hypertension 16 years prior, and there was no history of recent head trauma or infection. On his first admission, he was drowsy but well oriented, and his vital signs were stable except for his blood pressure (172/92 mmHg). He had signs of acute inflammation, as leukocytosis (24.82 × 10^3^ /μl) and increased level of hs-CRP (71.20 mg/L) were observed in the blood test. The blood test also revealed hyperglycemia (298 mg/dL). The results of the routine CSF study did not show any evidence of central nervous system infections.

**Fig. 1:**
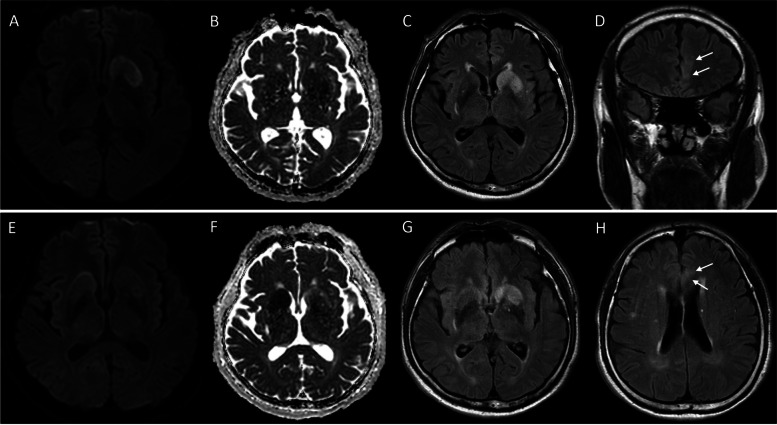
Brain MRI findings at 1st (**A-D**) and 2nd admission (**E-H**). On the first admission, the left caudate nucleus and putamen showed high signal intensity on DWI (**A**) and low signal intensity on the ADC map (**B**). Hyperintense lesions on FLAIR were detected in the left caudate nucleus and putamen (**C**). Acute lesions on the contralateral side were detected on the second admission (**E, F, G**). A slightly high signal intensity (arrow) on FLAIR was detected in the left medial frontal lobe (**D, H**)

Electroencephalography (EEG) results were normal, with no epileptic discharges. Brain magnetic resonance imaging (MRI) showed left caudate nucleus and putamen lesions, hyperintense on DWI, and hypointense on the ADC map (Fig. 1A, B). Furthermore, high-signal intensity lesions were detected on fluid attenuated inversion recovery (FLAIR) image, including the left caudate nucleus, putamen, and medial frontal lobe (Fig. 1C, D). However, the patient’s MR angiography results were normal.

Based on the brain MRI findings, we suspected acute ischemic stroke in the left basal ganglia. Anti-platelet and anti-epileptic agents (levetiracetam 1500 mg/day) were administered for ischemic stroke and focal seizure, respectively. On the seventh day of admission, he experienced hallucinations. He described that “something floats on the ceiling” and that “cartoon-like image pops-up”. We changed his drugs to sodium valproate and levetiracetam 3000 mg/day, for his symptoms did not improve. As his seizure occurred at a lower frequency, we reduced the dose of levetiracetam to 1500 mg/day, and he was discharged on the 14th day of admission.

Four days after discharge, the patient visited our emergency room again for another clonic seizure event. This time, the seizure involved his left upper extremity. In the emergency room, he was drowsy, but his vital signs were stable. Blood test results showed low levels of serum valproic acid (1.9μg/mL) and high levels of serum glucose (263 mg/dL). Brain MRI showed lesions in the caudate nucleus and putamen with a similar pattern to that of the last admission on DWI, ADC, and FLAIR, but on the opposite side (Fig. 1E, F, G). In addition, the same lesion previously observed on the left medial frontal lobe was identified on FLAIR image (Fig. 1H). The EEG did not reveal any abnormalities, while his tonic-clonic seizure with left eye deviation and left head turn continued. He also had symptoms of lip smacking and orofacial dyskinesia, which he was not aware of.

Given the suspicion of autoimmune encephalitis, we administered methylprednisolone 250 mg/day for 3 days. Also, the patient received anti-epileptic drugs (sodium valproate 1800 mg/day, levetiracetam 500 mg/day) to manage status epilepticus. As his symptoms improved after 3 weeks of medication, the patient was discharged. After discharge, his CSF test revealed the presence of anti-NMDA receptor antibodies, which confirmed anti-NMDAR encephalitis.

## Discussion and conclusions

This is a case of anti-NMDAR encephalitis with two separate focal seizures and stroke-like brain lesions on an MRI DWI/ADC map. The first event occurred on the right arm and hand, which then evolved into bilateral tonic-clonic seizures. Four days after he was discharged symptom-free, he had another seizure event involving his left arm and hand. On each admission, brain MRI showed caudate nucleus and putamen lesions on the contralateral side of the seizure. As DWI hyperintensity with decreased intensity on ADC map lesions was found on each admission, we suspected acute ischemic stroke followed by an acute symptomatic seizure. However, not only is focal seizure alone uncommon in stroke patients, but his symptoms recurred on the opposite side shortly after his original discharge. Therefore, stroke was less likely in this patient. Since anti-NMDA receptor antibodies were detected in the CSF, the patient was eventually diagnosed with anti-NMDAR encephalitis.

The caudate nucleus and putamen play roles in adjusting movements. When these areas are damaged, a patient exhibits symptoms of seizure and dyskinesia as our patient did [3–5]. When brain MRI shows high signal intensity on DWI and low signal intensity on the ADC map, it mostly suggests acute ischemic stroke and about 6% of the patients experience post-stroke seizures [6]. A study of 3050 stroke patients reported that the incidence of caudate nucleus stroke was 1% of all patients and only 12% of them showed bilateral involvement [7]. In addition, in some cases, a few patients with bilateral caudate nucleus stroke have variants of Circle of Willis [8]. In this case, the patient showed bilateral basal ganglia brain lesions on brain MRI without any vascular abnormalities. Furthermore, the fact that the brain lesion did not occur simultaneously, but consecutively with a short time interval, is also incompatible with past reports.

A variety of conditions can occur in bilateral basal ganglia lesions, such as genetic disorders, metabolic disorders, infections, intoxication, and autoimmune encephalitis [9]. As the patient did not have any history of genetic disorders, infections, or intoxications, we sought other causes. It was also less likely that hyperglycemia provoked the brain lesions, for his serum glucose was measured lower than 300 mg/dL and hyperglycemic encephalopathy usually causes unilateral brain lesion [9]. On the other hand, some autoimmune encephalitis affect bilateral basal ganglia on MRI and accompany seizure and dyskinesia [9]. As the patient showed similar manifestations above, we assumed that he was afflicted with autoimmune encephalitis. His CSF tested positive for anti-NMDA receptor antibodies.

According to the largest case series, approximately half (55%) of anti-NMDAR encephalitis patients have brain abnormalities on MRI, and only 5% of them were in the basal ganglia [1]. Recently, some reports have revealed the involvement of the basal ganglia by T2-weighted imaging, PET-computed tomography (CT), and single-positron emission computerized tomography (SPECT) [2, 5]. There were even cases that affected the bilateral basal ganglia [10, 11]. This case was clinically significant in two aspects. First, the bilateral basal ganglia lesions did not develop simultaneously but developed with a short time interval. Second, the abnormalities were discovered using the DWI/ADC map.

In previous studies, the basal ganglia were not considered the original seizure focus. Rather, it is largely understood to be involved in the propagation circuit of seizures. However, a recent study identified that caudate nucleus can generate seizure. The patient complained symptom of seizures with head turn to the left and hyperkinetic movements of trunk and limbs [3]. In another study, an anti-NMDAR encephalitis patient with seizure showed hyperperfusion on the basal ganglia on SPECT image, indicative of a focal seizure arising from the basal ganglia [5]. We assumed that the basal ganglia was the primary seizure focus as our patient showed similar semiologies to the cases above. Furthermore, cortico-striatal synchronization is thought to be responsible for the high signal intensity of the left medial frontal lobe found on the FLAIR image [12].

In conclusion, when a patient with focal seizure develops bilateral brain lesions sequentially as described above, autoimmune encephalitis should be considered, and CSF antibody testing may help with the diagnosis. In addition, we believe that the DWI/ADC map could help diagnose patients in the early course of autoimmune encephalitis.

## Data Availability

All data and material supporting our findings are contained within the manuscript.

## Abbreviations

NMDAR: N-methyl-D-aspartate receptor
MRI: Magnetic resonance imaging
FLAIR: Fluid-attenuated inversion recovery
DWI: Diffusion-weighted imaging
ADC: Apparent diffusion coefficient
CSF: Cerebrospinal fluid
^18^F-FDG-PET: ^18^F-fluorodeoxyglucose-positron emission tomography
EEG: Electroencephalography
CT: Computed tomography
SPECT: Single-positron emission computed tomography
